# Cement-augmented locked plate fixation proximal humerus fractures in elderly patient: a systematic review and meta-analysis

**DOI:** 10.1186/s12891-024-07502-1

**Published:** 2024-05-10

**Authors:** Dong-Yang Li, Kun Zhang

**Affiliations:** Department of Orthopedic Trauma, Honghui Hospital, Xi’an Jiaotong University, No. 555 Youyi East Road, Xi’an, Shaanxi Province 710054 P.R. China

**Keywords:** Cement, Proximal humerus fracture, Augmentation, Locked plate, Meta-analysis

## Abstract

**Background:**

This systemic review and meta-analysis aimed to evaluate the clinical outcomes of proximal humeral fracture in elderly patient fixation using locked plate with or without cement augmentation.

**Methods:**

The databases of PubMed, Embase, and Cochrane Library were searched in August 2023 for literature comparing the clinical outcomes of patients with PHFs treated with locked plate alone and locked plate augmented with cement. Data describing study design; level of evidence; inclusion criteria; demographic information; final follow-up; revision rate; implant failure rate; avascular necrosis rate; total complication rate; constant score; and disability of arm, shoulder, and hand (DASH) score were collected.

**Results:**

Eight studies (one randomized-controlled trial and seven observational studies), involving 664 patients, were identified. Compared with locked plates alone, using cement-augmented locked plates reduced the implant failure rate (odds ratio (OR) = 0.19; 95% confidence interval (CI) 0.10–0.39; *P* < 0.0001) and total complication rate (OR = 0.45; 95% CI 0.29–0.69; *P* = 0.0002) and improved DASH scores (mean difference (MD) = 2.99; 95% CI 1.00–4.98; *P* = 0.003). However, there was no significant difference in clinical outcomes, including revision rate, avascular necrosis rate, and constant score.

**Conclusion:**

In this review and meta-analysis, fixation of the PHFs in elderly patients using locked plates with or without cement augmentation has no significant difference in revision rate, but the implant failure and total complication rates may be lesser on using the cement-augmented locked plate for fixation than on using a locked plate alone. Good results are expected for most patients treated with this technique.

**Trial registration:**

The Preferred Reporting Items for Systematic Reviews and Meta-Analyses (PRISMA)21 guidelines were followed to conduct this systematic review and meta-analysis and was registered as a protocol in PROSPERO (CRD42022318798).

**Supplementary Information:**

The online version contains supplementary material available at 10.1186/s12891-024-07502-1.

## Introduction

Fractures of the proximal humerus account for 5% of all fractures and are the second most common fractures of the upper extremity after distal radius fractures [1]. While some proximal humeral fractures (PHFs) are either non-displaced or minimally displaced and can be treated non-operatively with good functional results, complex PHFs often occur in older adults, especially women, and surgery is usually required [2, 3].

Numerous surgical techniques for the treatment of complex PHFs, such as percutaneous techniques, intramedullary nailing, plating, and arthroplasty, have been established [4, 5]. Among these, open reduction and internal fixation with locked plates have gained wide acceptance and have shown good clinical outcomes [6–8]. But still not reached desirable levels, the reported complication rates and revision rates remain high [9–11]. In a multicenter, prospective study by Brunner et al. [12], 13.8% of cases required revision surgery following mechanical failure. In another study by Königshausen et al., complication rates of up to 23% were reported [13]. The presence of osteoporotic bone in the older patient population makes screw anchorage difficult. A lack of medial support has been identified as the main cause of treatment failure [14, 15]. Considering the above-mentioned factors, the ideal implant should not only make the construct flexible enough to unload the bone-implant interface but also rigid enough to minimize fracture movements [16].

To enhance stability in internal fixation and to avoid implant failure requiring revision surgery, several techniques have been tested. (For healthy patients under 60 years of age, plates are usually used alone. For patients over 60 years of age with known osteoporosis and decreased bone mineral density, fracture augmentation may be an option.) [14]. Autograft and allografts with the purpose of addressing the need for medial support and fill the void after osteoporotic fractures. Although it has achieved good clinical results, its limitations limit its wide use.^30^ Mechanical devices can also be used for augmentation, despite different design, share similar biomechanical principles. In the literature, two systems have been used in the setting of PHFs: (1) The Da Vinci System or “triangular block bridge”, and (2) The Proximal Humerus Cage or “intramedullary cage”. These implants aim to provide structural support to the humeral head and fill the metaphyseal void. The most commonly described techniques were, in fact, reinforcing the screw-bone interface with cement. There are many kinds of bone cement, Polymethylmethacrylate (PMMMA), calcium phosphate and calcium sulfate, their respective characteristics are different [18, 19]. Cement augmentation technique is also slightly different around the word. The most commonly employed technique involves the utilization of cannulated screws for fracture fixation to the plate, then prefilled syringes with PMMA were used to augment cannulated screw with 0.5 to 1 mL of cement for reduction the risk of screw cut-out [14, 17, 19]. Several studies also reported the technique of filling the cement at metaphysical medullary canal around the fracture site [19].

To analyze the available data, a recent systematic review examined the use of augmentation in the treatment of PHFs. Marongiu et al. [19] conducted a systematic review of 10 studies that reported the clinical application of cement, bone substitutes, and metallic devices for fracture augmentation in patients with osteoporotic PHFs. The studies included in that systemic review were not sufficient enough to conduct a meta-analysis at that time. Following the publication of the systematic review, two studies comparing fixation with cement-augmented locked plates to locked plates alone in a larger sample size were published.

This review and meta-analysis aimed to analyze the clinical outcomes of cement for fracture augmentation with PHFs in elderly patients.

## Materials and methods

The Preferred Reporting Items for Systematic Reviews and Meta-Analyses (PRISMA)21 guidelines were followed to conduct this systematic review and meta-analysis and was registered as a protocol in PROSPERO (CRD42022318798).

(1) Inclusion and exclusion criteria were as follows: 1) Randomized controlled trials (RCTs) or observational studies (OSs), including cohort and case-control studies;2) Comparing the clinical outcomes of using cement-augmented locked plate fixation and without cement-augmented in the management of proximal humerus fractures in elderly patients (Patients over 60 years of age with significant displacement and severe osteoporotic requiring open reduction and internal fixation); 3) a minimum means radiological and clinical follow-up period of 6 months; 4) postoperative surgical data, and functional and radiological outcome data are available. The exclusion criteria were biomechanical studies, computational and finite element analyses, and other nonclinical applications. Moreover, case reports and gray literature were also excluded.

(2) Primary outcomes: Revision and implant failure rates (implant failure in this meta-analysis included loss of reduction, fracture collapse, screw penetration, or screw back-out, as defined and reported by the respective authors).

(3) Secondary outcomes: Avascular necrosis rate, total complication rate (total complications included implant failure, avascular necrosis, wound infection, persistent pain, nerve injury, plate subacromial impingement, and nonunion), the DASH score, and the constant score.

### Search Strategy

We searched the databases of PubMed, Embase, and Cochrane Library according to the Cochrane Handbook for Systematic Reviews of Interventions using the following terms: (humeral fracture proximal) AND (bone substitutes OR augmentation OR hydroxyapatite OR cement OR polymethylmethacrylate (PMMA) OR calcium sulfate OR calcium phosphate). The search period was from database creation to August 2023. There were no restrictions in the search process.

### Data extraction and quality analysis

Two professional reviewers extracted the data and evaluated the quality of the text in the included articles. Disagreements between the two reviewers were resolved by consensus. The recommendations by Cochrane Handbook for Systematic Reviews of Interventions was used to evaluate the quality of the RCT, including sufficient random sequence generation, allocation concealment, blinding, incomplete result data, selective reporting bias, and other biases. The MINORS criteria [20] was used to evaluate the quality of the OSs. Items were scored as 0 for not reported, 1 for reported but inadequate, and 2 for reported and adequate. For a comparative study, the ideal score was 24.

### Statistical analysis

Review Manager (RevMan, The Cochrane Collaboration, London, United Kingdom) version 5.3 was used for the statistical analysis. We used odds ratios (OR) and mean differences (MD) to present count data and continuous variables, with 95% confidence intervals (CIs). When the statistical heterogeneity between studies was low (*P* > 0.1, *I*^*2*^ < 50%), the fixed-effects model was used for analysis. In case of high statistical heterogeneity between studies (*P* < 0.1, *I*^*2*^ > 50%), the possible sources of heterogeneity and possible interference factors were analyzed [21]. If there was only statistical heterogeneity but no clinical heterogeneity, a random-effects model was used to pool the data. Statistical significance was set at *P* < 0.05.

## Results

### Literature search

**Fig. 1:**
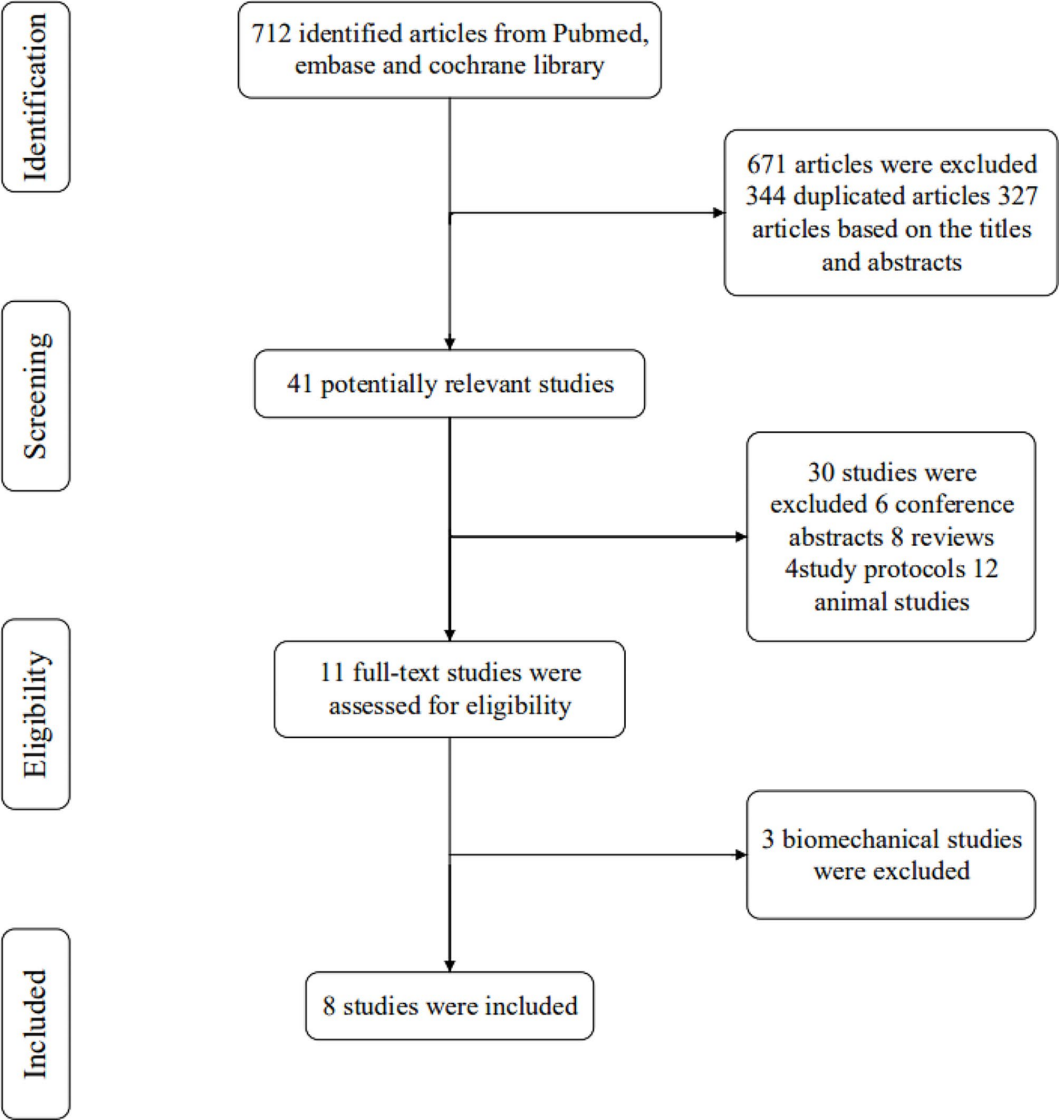
Flowchart of included studies

The initial database search yielded 712 studies out of which 344 duplicates were excluded. After the preliminary screening, 327 articles were further excluded based on their titles and abstracts. From the remaining 41 studies, the reviewers excluded reviews, protocols, biomechanical studies, and animal studies as per the inclusion and exclusion criteria. Finally, eight studies were included, comprising one RCT and seven case-control studies [22–29]. A flowchart of the process is illustrated in Fig. 1.

### Baseline information of the included studies

The eight studies had a total of 664 patients at baseline; at study completion, only 635 patients were included in the data analysis (334 patients treated with locked plate alone and 301 with cement-augmented locked plate). Baseline information of the included studies is presented in Table 1.

**Table 1:** Baseline information of included studies

Author	Year	Title	Study Design	Total Score
Foruria	2021	Proximal humeral fracture locking plate fixation with anatomic reduction, and a short-and-cemented-screws configuration, dramatically reduces the implant related failure rate in elderly patients	Retrospective Cohort	19
Hakimi	2021	Angle-stable polyaxial locked plating with and without polymethylmethacrylate cement augmentation for proximal humeral fractures in elderly	Retrospective Cohort	18
Siebenbürger	2019	Screw-tip augmentation versus standard locked plating of displaced proximal humeral fractures: a retrospective comparative cohort study	Retrospective Cohort	18
Katthagen	2018	Cement augmentation of humeral head screws reduces early implant-related complications after locked plating of proximal humeral fractures	Prospective non-randomized	16
Egol	2012	Fracture site augmentation with calcium phosphate cement reduces screw penetration after open reduction internal fixation of proximal humeral fractures	Retrospective Cohort	16
Liu	2011	Use of a proximal humeral internal locking system enhanced by injectable graft for minimally invasive treatment of osteoporotic proximal humeral fractures in elderly patient	Retrospective Cohort	18
Lee	2009	Prognostic factors for unstable proximal humeral fractures treated with locking-plate fixation	Retrospective Cohort	15

### Quality Assessment

The RCT used sealed hidden envelopes for allocation but did not specifically mention the blinding that was followed [26]. On quality assessment of the OSs, three had a total MINORS score of 18 [23, 24, 29]; two studies by Katthagen et al. and Egol et al. had a total MINORS score of 16 [25, 27]. Among the other included studies, the study by Foruria et al. had the highest total MINORS score of 19, whereas the lowest total MINORS score of 15 was recorded in a study by Lee et al. [22, 28] (Table 2).

**Table 2:** MINORS bias score

Author	Year	Title	Study Design	Total Score
Foruria	2021	Proximal humeral fracture locking plate fixation with anatomic reduction, and a short-and-cemented-screws configuration, dramatically reduces the implant related failure rate in elderly patients	Retrospective Cohort	19
Hakimi	2021	Angle-stable polyaxial locked plating with and without polymethylmethacrylate cement augmentation for proximal humeral fractures in elderly	Retrospective Cohort	18
Siebenbürger	2019	Screw-tip augmentation versus standard locked plating of displaced proximal humeral fractures: a retrospective comparative cohort study	Retrospective Cohort	18
Katthagen	2018	Cement augmentation of humeral head screws reduces early implant-related complications after locked plating of proximal humeral fractures	Prospective non-randomized	16
Egol	2012	Fracture site augmentation with calcium phosphate cement reduces screw penetration after open reduction internal fixation of proximal humeral fractures	Retrospective Cohort	16
Liu	2011	Use of a proximal humeral internal locking system enhanced by injectable graft for minimally invasive treatment of osteoporotic proximal humeral fractures in elderly patient	Retrospective Cohort	18
Lee	2009	Prognostic factors for unstable proximal humeral fractures treated with locking-plate fixation	Retrospective Cohort	15

### Outcome measures

#### Primary outcomes

##### Revision rate

**Fig. 2:**
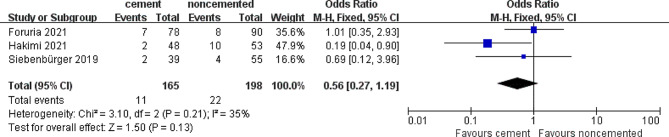
Forest plot of revision rate in cement and noncemented groups

The revision rate following surgery was the most important outcome analyzed in this systemic review. Three of the eight studies reported revision rates caused by varied reasons, as enumerated in Fig. 2. Owing to the low heterogeneity, meta-analysis using the fixed-effects model did not reveal any significant difference (OR = 0.56; 95% CI 0.27–1.19; *P* = 0.13) in the revision rate between the locked plate only and locked plate augmented with cement groups. We conducted a sensitivity analysis after removing the most weighted study by Hakimi et al. [23], which showed no significant difference in the number of revision surgeries between the locked plate only and the locked plate augmented with cement groups (OR = 0.91; 95% CI 0.37–2.24; *P* = 0.84), with low heterogeneity (*I*^*2*^ = 0%).

Foruria et al. reported the various causes for revision surgery in each study group; while most revisions surgeries were due to loss of fixation and plate mechanical interference, the ones due to avascular necrosis were rare [22]. In a study by Katthagen et al., where only the cement-augmented locked plate group was analyzed, six out of 24 patients (25%) underwent early arthroscopic revision surgery, owing to a limited postoperative range of motion, despite high functional expectations [25]. Although Egol et al. reported revision surgery in 11 out of 92 patients (12%), their subgroups could not be delineated [27]. Revision rates were not reported in the remaining three studies either due to shorter follow-up times or successful surgeries.

### Implant failure rate

**Fig. 3:**
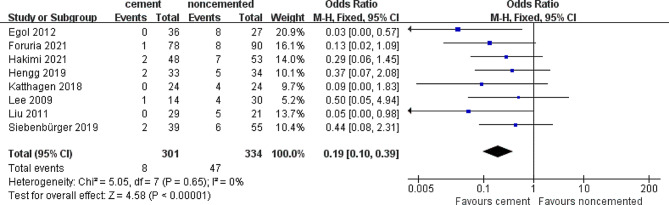
Forest plot of implant failure rate in cement and noncemented groups

The implant failure rate, another important outcome analyzed in this systemic review, was reported by eight studies. There were 47 reported implant failures in 334 (14%) patients treated with locked plates alone. Only 8 implant failures were recorded in 301 (2.6%) patients treated with the cement-augmented locked plate. There was a statistically significant decrease in the OR for implant failure in the locked plate augmented with cement group compared with that in the locked plate only group (OR = 0.19; 95% CI 0.10–0.39; *P* < 0.05), with low heterogeneity (*I*^*2*^ = 0%), as shown in Fig. 3. This suggests that the reliability of fixation using cement-augmented locked plate is higher than that of the locked plate alone.

### Secondary outcomes

#### Avascular necrosis rate

**Fig. 4:**
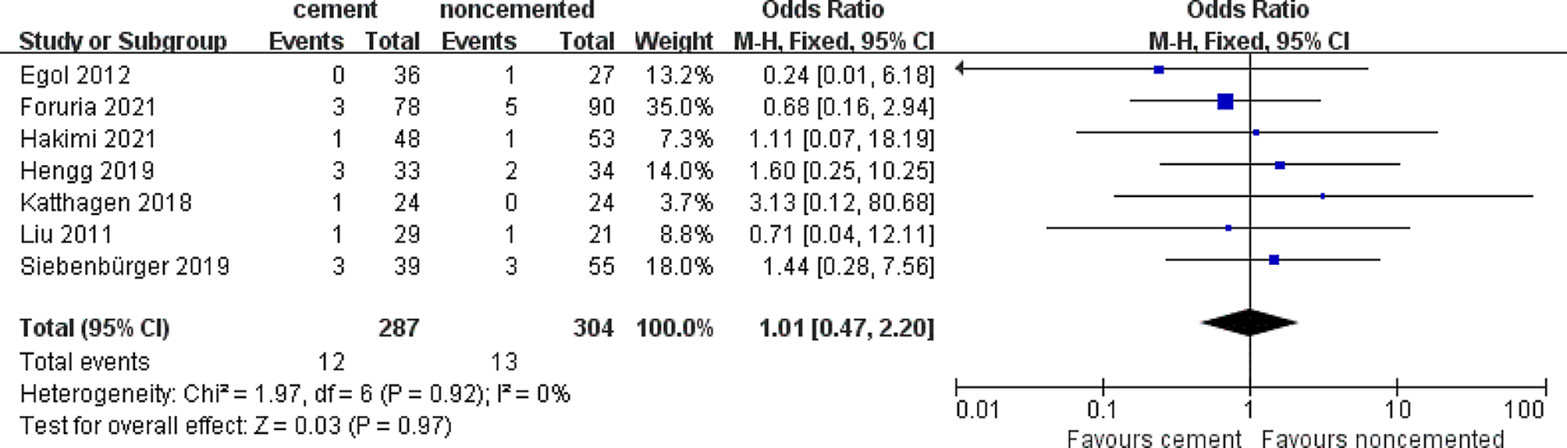
Forest plot of avascular necrosis rate in cement and noncemented groups

Seven studies assessed the difference in the avascular necrosis rate. There were 13 reported cases of avascular necrosis in 304 (4.2%) patients treated with the locked plate alone, whereas 12 cases of avascular necrosis were recorded in 287 (4.3%) patients treated with cement-augmented locked plate, as depicted in Fig. 4. We found no significant difference in the incidence of avascular necrosis between the locked plate only and the locked plate augmented with cement groups (OR = 1.01; 95% CI 0.47–2.20; *P* = 0.97).Similarly, the heterogeneity in the avascular necrosis rate was low (*I*^*2*^ = 0%). In other words, cement augmentation may not increase the risk of avascular necrosis.

### Total complication rate

**Fig. 5:**
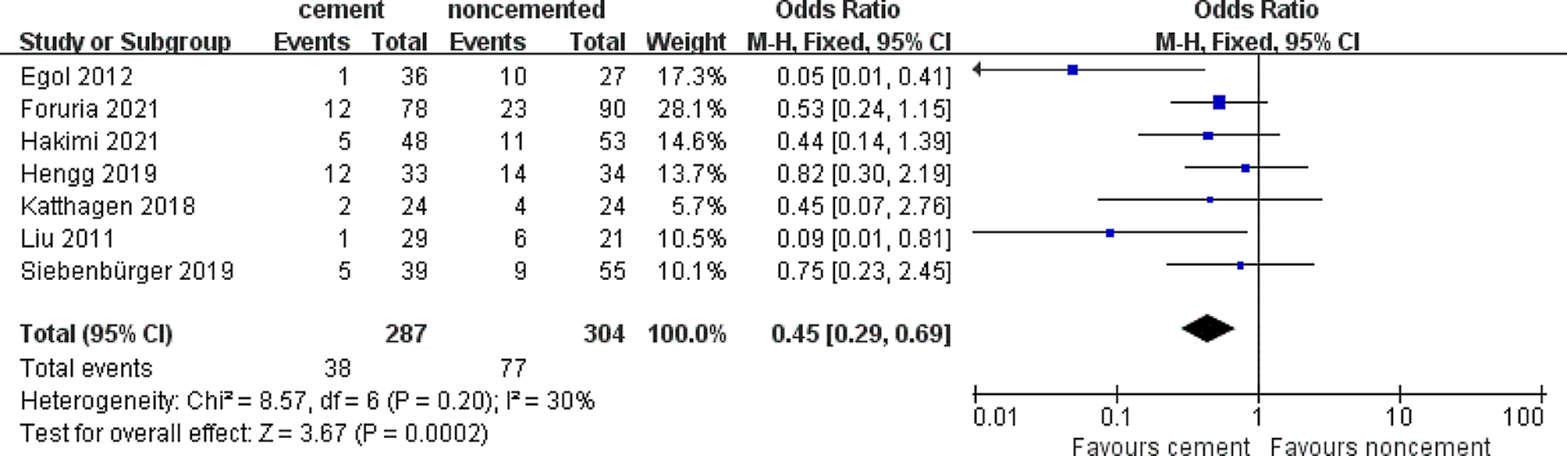
Forest plot of total complications rate in cement and noncemented groups

Seven out of the eight studies reported the total complication rates in each study group. There were 77 complications reported in 304 (25%) patients treated with the locked plate alone, while 38 complications were noted in 287 (13.2%) patients treated with the cement-augmented locked plate. Owing to the low heterogeneity (*I*^*2*^ = 30%), a meta-analysis using the fixed-effects model was conducted, which revealed that fixation with cement-augmented locked plate is associated with a decrease in the total complication rate (OR = 0.45; 95% CI 0.29–0.69; *P* = 0.05) (Fig. 5).

Lee et al. [28] reported the total complication rate in 44 patients, but the number of complications recorded in each group was not specified. Of the 44 patients, 9 (20%) had postoperative complications, with a loss of fixation in five, adhesive capsulitis in three, and deep infection in one.

### DASH score

**Fig. 6:**
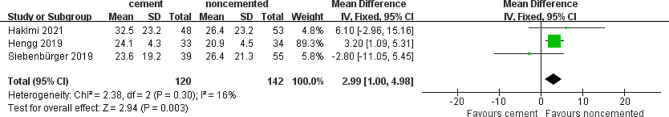
Forest plot of DASH score in cement and noncemented groups

Three studies used the DASH score to measure functional recovery of the upper limb. We found a difference in heterogeneity (*I²=*16%) between the locked plate only and the locked plate augmented with cement groups (MD = 2.99; 95% CI 1.00–4.98; *P* = 0.05) as shown in Fig. 6. The meta-analysis using the fixed-effects model concluded that patients in the cement-augmented locked plate group may have better functional recovery of the upper limbs. We conducted a sensitivity analysis after removing the most weighted study, by Hengg et al. [26], which showed no significant difference in DASH score between the locked plate only and the locked plate augmented with cement groups (MD = 1.44; 95% CI 7.27–10.16; *P* = 0.75) However, the heterogeneity was relatively high (*I*^*2*^ = 51%), indicating that the conclusion may not be reliable.

### Constant score

**Fig. 7:**
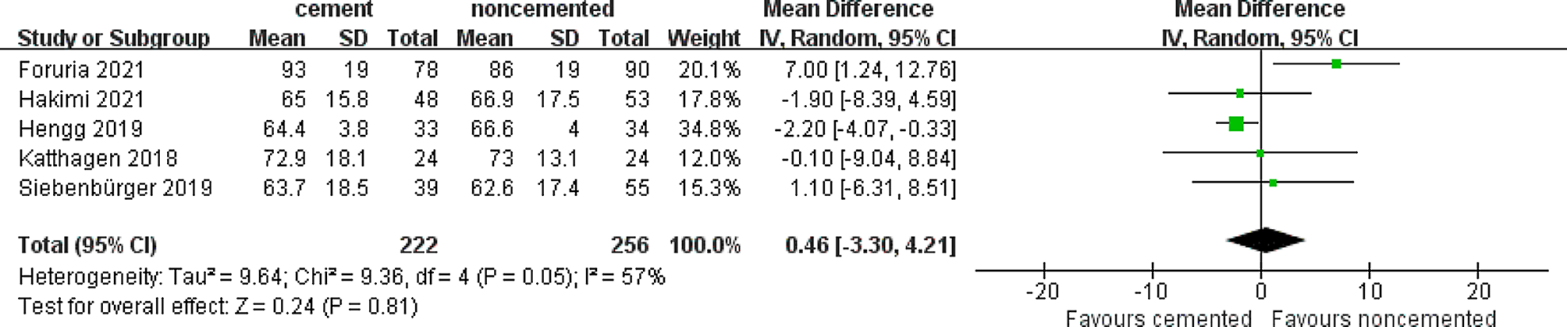
Forest plot of constant score in cement and noncemented groups

Five studies assessed the difference in constant scores between the locked plate only (256 patients) and cement-augmented locked plate groups (222 patients). The heterogeneity was high (*I*^*2*^ = 57%). After verification, no significant clinical heterogeneity was found between the two groups, probably because of differences in the time of postoperative evaluation. The pooled results from the random-effects model suggested no significant difference in the constant score between the two groups (MD = 0.46; 95% CI 3.30–4.21; *P* = 0.81), as shown in Fig. 7. Similarly, the difference was not significant (MD = 1.94; 95% CI 2.36–6.24; *P* = 0.38) after removing the most weighted study by Hengg et al. and the heterogeneity was low (*I*^*2*^ = 34%) [26].

## Discussion

The main goal of fracture augmentation is to provide mechanical support to osteoporotic bones. In otherwise healthy patients under 60 years of age, plates are usually used alone. The decision to use fracture augmentation is an option for patients over 60 years of age with known osteoporosis and reduced bone density [17] The most commonly described techniques are auto- and allografting. The concerns about possible autograft-related morbidity in the donor, their availability, and the associated high costs of allograft fixation are problematic to its extensive use [30]. The application of cement has been proposed as an alternative for the augmentation of osteoporotic PHFs in order to enhance screw anchorage and increase the primary stability of locked plates for displaced PHFs [17, 18]. Although good to excellent outcomes of reinforcing the screw-bone interface with cement have been reported in the literature, these options still lack long-term follow-up and large sample comparative studies.

Our meta-analysis included eight papers published from 2009 to 2021 that evaluated the clinical outcomes of 635 older patients with PHFs. The major findings of this meta-analysis are as follows: (1) locked plate with or without cement augmentation for PHFs has the same revision rates, but compared with fixation with locked plate alone, fixation with the cement-augmented locked plate could reduce implant failure and total complication rates; (2) similarly, the cement-augmented locked plate does not increase the risk of avascular necrosis compared with the locked plate alone; (3) cement augmentation effects on clinical functional recovery of the upper limb remain controversial.

According to this meta-analysis, three of the eight studies reported two groups of revision rates for various reasons. There were 22 reported revisions in 198 (11%) patients treated with the locked plate alone, and 11 reported revisions in 165 (6.6%) patients treated with the locked plate augmented with cement. Though cement augmentation can effectively reduce revision rates, the effect is not statistically significant. It is important to note that the implantation of bone cement is generally considered to increase the difficulty and failure rate of secondary revision surgery, in particular arthroplasty. However, Foruria et al. showed that removal of cement augmented screws was technically easy, provided all screws heads had been cleared of cement during the index procedure [22]. A reduction in the rate of revision is generally associated with a significant reduction in the rate of implant failure. However, in this review, although implant failure rates declined significantly, revision rates did not. This may be related to different expectations from the surgery and shorter follow-up time. In terms of implant failure and total complication rates, treatment with cement-augmented locked plate reduced the implant failure rates from 14 to 2.6% and the total complication rates from 25 to 13%. A locked plate is usually the first choice for the fixation of displaced PHFs. However, owing to osteoporosis in the affected elderly population and the associated difficult screw anchorage, the complication rates are still high. Researchers from the Mayo Clinic reported a 44% total complication rate and a 35% implant failure rate in 2020 [31]; similar results have been widely reported over the years [10, 32, 33]. Various biomechanical and clinical investigations have been performed to achieve stable implant anchorage in PHFs to enhance the stability in internal fixation and avoid implant failure requiring revision surgery. Kwon et al. conducted biomechanical evaluation with calcium phosphate cement in cadaveric limbs and found that supplementation with calcium phosphate cement led to significant improvements in the mechanical performance of internal fixation [34]. Röderer et al. showed that screw augmentation could compensate for osteoporotic bones [17]. Some researchers have reported less movement at the interface between the bone and implant in cement-augmented locked plate osteosynthesis [17, 27, 35]. Almost all in-vitro studies have shown that cement augmentation increases the mechanical strength of the fixation. Our meta-analysis demonstrated that the biomechanical benefits of cement augmentation are clinically applicable without involving additional complications, which could greatly reduce the implant failure and total complication rates. Polymethylmethacrylate (PMMA), the most widely used bone cement, can reach temperatures as high as 100 °C during the polymerization phase, which could potentially cause bone and cartilage necrosis [36]. The results of our meta-analysis showed a 4.2% humeral head necrosis rate in the group fixated with locked plate alone and 4.1% in the group fixated with cement-augmented locked plate. This finding suggests that PMMA does not increase the probability of humeral head necrosis, which is consistent with the view of some researchers [37, 38]. In addition, it should be noted that humeral head necrosis is not only associated with PMMA but may also be associated with the degree of fracture comminution. However, existing clinical studies do not include these data, and further analysis of the impact of cement augmentation on the rates of humeral head necrosis cannot be done.

This meta-analysis used the DASH score and constant score to assess postoperative clinical function. Interestingly, the two criteria produced inconsistent results. On pooling the data from three studies that used the DASH score to assess functional outcomes, it was concluded that cement augmentation may be detrimental to functional recovery [23, 24, 26]. Data from five studies using the constant score to assess functional outcomes did not reveal any significant difference between the cement augmentation group and the locked plate only group; however, the heterogeneity was high [22–26]. This is due to the variable duration of follow-up in the individual studies when calculating the functional score (ideally the studies should have the same follow-up duration). A subgroup analysis can eliminate the heterogeneity seen in this case, provided there are enough studies to be included. Therefore, undertaking further studies could clarify the impact of cement augmentation on functional rehabilitation.

This study has several limitations. First, seven of the included studies were OSs, and only one was an RCT. While the results presented are promising, more RCTs are needed to determine the true efficacy of cement augmentation in the management of PHFs treated with locked plates. Second, the follow-up times in the RCT and OSs included in this review were inconsistent, and the internal design of each study had varying degrees of inadequacy. These factors may affect the authenticity of the results. Third, the potential risk of leakage with cement use has not been mentioned in any of the eight articles, which may underestimate the adverse effect of cement augmentation [39]. Similarly, the lack of evaluation of radiographic parameters is a significant limitation. Further high-quality RCTs with longer follow-up times, unified measurement standards, and unified radiographic parameters are needed for generating better evidence.

## Conclusions

In this review and meta-analysis, fixation of the PHFs in elderly patients using locked plates with or without cement augmentation has no significant difference in revision rate, but the implant failure and total complication rates may be lesser on using the cement-augmented locked plate for fixation than on using a locked plate alone. Good results are expected for most patients treated with this technique.

### Electronic supplementary material

Below is the link to the electronic supplementary material.

Supplementary Material 1

## Data Availability

All data generated or analysed during this study are included in this published article.

## Abbreviations

PHF: proximal humerus fracture
DASH: Disability of Arm Shoulder and Hand
RCT: randomized controlled trial
OS: observational study
PMMA: Polymethylmethacrylate
CPC: Calcium Phosphate Cement
CSC: Calcium Sulfate Cement
OR: odds ratio
MD: mean difference
CI: confidence interval
